# Kai-Xin-San, a Chinese Herbal Decoction, Accelerates the Degradation of *β*-Amyloid by Enhancing the Expression of Neprilysin in Rats

**DOI:** 10.1155/2020/3862342

**Published:** 2020-02-21

**Authors:** Na Wang, Yongming Jia, Bo Zhang, Yan Li, Ghulam Murtaza, Shuming Huang, Xuewei Liu

**Affiliations:** ^1^Department of Neuropharmacology, College of Pharmacy, Qiqihar Medical University, Qiqihar 161006, China; ^2^Department of Neuroscience, Institute for Traditional Chinese Medicine, Heilongjiang University of Chinese Medicine, Harbin 150040, China; ^3^Institute of Integrative Traditional and Western Medicine, Medical College, Yangzhou University, Yangzhou 225009, China; ^4^Department of Pharmacy, COMSATS University Islamabad, Lahore Campus 54000, Lahore, Pakistan

## Abstract

This study aimed to investigate the mechanisms of Kai-Xin-San (KXS, a famous Chinese herbal decoction used to treat amnesia) on the degradation of A*β* and further elucidate the mechanism of KXS on A*β*-induced memory dysfunction. After pretreatment with KXS (1.08 g/kg/day) for two weeks, A*β*_42_ (2 *μ*L, 200 *μ*M) was injected into rat hippocampus to induce cognitive dysfunction. Morris water maze (MWM) test was developed to evaluate cognitive performance in rats. Hippocampal neurons were observed by histological staining using Hematoxylin-Eosin (HE) methods. Levels of exogenous A*β*_42_, which was injected into the hippocampus, were continually measured through a special Enzyme-linked immunoassay (ELISA) kit to observe the catabolic process of A*β* in the brain. Similarly, A*β* degradation in PC12 cells was also investigated using the ELISA kit. The expressions of A*β* degeneration enzymes, including neprilysin (NEP), angiotensin-converting enzyme (ACE), and endothelin-converting enzyme (ECE), were detected by western blotting to elucidate A*β* reduction mechanism. Our results showed that KXS prevented A*β*_42_-induced cognitive impairment and attenuated hippocampus neuronal damage caused by A*β*_42_. Moreover, KXS could accelerate A*β*_42_ degradation in A*β*_42_ injected rats. Furthermore, NEP, an A*β* degradation enzyme, was increased in the hippocampus while ECE and ACE, other two A*β*-degrading enzymes, were not changed. KXS accelerated A*β* degradation in PC12 cells. Our findings revealed that KXS facilitated the degradation of A*β*_42_ by increasing the expression of NEP in rat hippocampus. By reducing the A*β* burdens, KXS protected hippocampal neurons, leading to the improvement of cognitive function in rats.

## 1. Introduction

Alzheimer's disease (AD), a neurodegenerative disease associated with symptoms of cognitive dysfunction, is mainly characterized by the presence in the brain of senile plaques due to amyloid beta (A*β*) peptides deposits and neurofibrillary tangles [1]. An abnormal A*β* increase is considered the first sign indicating AD development [2]. A*β* homeostasis in the brain is governed by its production and clearance mechanisms. Under normal conditions, A*β* in the brain is constantly produced from an amyloid precursor protein (APP), which is sequentially cleaved by *β*- and *γ*-secretases, and mostly catabolized by A*β*-degrading enzymes (ADEs) including neprilysin (NEP), angiotensin-converting enzyme (ACE), and endothelin-converting enzyme (ECE). A*β* degradation by ADEs, the main modality to avoid cerebral A*β* accumulation, plays a central role in sustaining A*β* normal levels [3]. The imbalance between its production and clearance can lead to excessive A*β* accumulation in the brain, causing AD typical pathological cascade reactions. Studies have demonstrated that anabolic increase of A*β* was rarely observed in sporadic AD which accounts for the overwhelming percentage of AD [4]. Therefore, the importance of A*β* clearance in AD pathogenesis has been raised, and A*β* catabolic mechanism became the new therapeutic target.

Ding-Zhi-Wan, a famous herbal formula of traditional Chinese medicine that was formerly reported in Chinese ancient book (Bei Ji Qian Jin Yao Fang) by Sun Simiao, has another name as Kai-Xin-San (KXS) in (Tai Ping Hui Min He Ji Ju Fang) [5]. Kai-Xin-San (KXS), a famous herbal formula of traditional Chinese medicine, consists of Radix Ginseng (*Radix Ginseng C.A. Meyer*), Poria (*Poria cocos F.A. Wolf*), Polygalae (*Polygala tenuifolia Wild*) and Acorus (*Acorus tatarinowii rhizome*) with dosage proportion of 3 : 3 : 2 : 2, has been used to treat amnesia for thousands of years in China. Indeed, KXS improves learning and memory in experimental AD studies and the mechanisms were correlated with multiple effects [5, 6], including reduced A*β* toxicity, neuroprotection, and neurites regeneration improvement. Similarly, our previous studies demonstrated that KXS ameliorates neuron loss and cognitive dysfunction induced by A*β* in vivo [7].

In other words, those studies usually focus on the symptom-alleviating effects of KXS on a neuropathological cascade of events caused by deposition of A*β*. In addition, our previous study showed that KXS could upregulate insulin-degrading enzyme (IDE); however, whether KXS could involve other ADEs mediated A*β* catabolic pathway has not been exhaustively illuminated. Therefore, our study aimed to further evaluate the mechanisms from the catabolic pathway of KXS ability on ameliorating AD.

## 2. Materials and Methods

### 2.1. Materials

Herbs of Ginseng (Renshen; the root of *Panax ginseng C. A. Mey.*), Poria (Fuling; sclerotium of *Poria cocos (Schw.) Wolf*), Polygalae (Yuanzhi; the root of *Polygala tenuifolia Willd*. or *Polygala sibirica* L.), and Acorus (Shichangpu; the root of *Acorus tatarinowii Schott.*) were purchased from Harbin Tongrentang Drug Company (Harbin, China) in Heilongjiang Province and were authenticated by Dr. Shuming Huang. Thiorphan was purchased from Abcam Company. Primary antibodies against rabbit NEP, ECE, and ACE were purchased from Santa Cruz Technology (California, USA). Monoclonal antibody against mouse *β*-actin and the enzyme horseradish peroxidase (HRP)-linked secondary antibodies were purchased from Beyotime Institute of Biotechnology (Beijing, China). Other reagents and solvents were of analytical grade and were commercially available.

### 2.2. Animals and Experimental Protocol

A total of 150 male Wistar rats (180–220 g) were provided by the Laboratory Animal Center of Heilongjiang University of Chinese Medicine (Harbin, China). Animals were housed in an animal laboratory with a temperature of 22 ± 2°C, 60 ± 3% humidity, and 12 h dark/light cycles with ad libitum access to rat chow and tap water. The animal facilities and protocols were approved by the Institutional Animal Care and Use Committee of the Heilongjiang University of Chinese Medicine. All procedures were in accordance with the National Institute of Health's guidelines regarding the principles of animal care [3].

**Figure 1:**
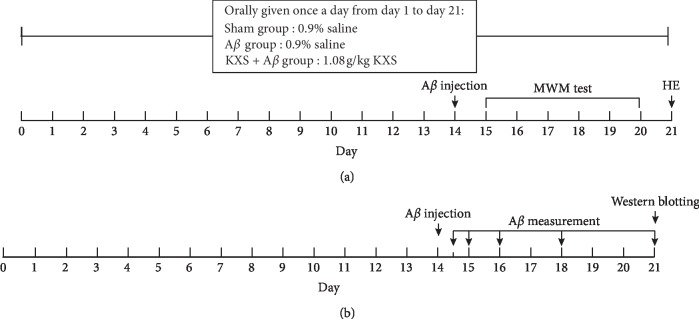
Two experimental protocols. (a) was developed for the MWM test and HE staining. At day 14, A*β* was injected into rat hippocampus in the A*β* group as well as the KXS + A*β* group, whereas 0.9% saline was injected in the sham group. After the MWM test from day 15 to day 20, HE was developed on day 21. (b) was developed for A*β* measurement and western blot for NEP, ACE, and ECE. ELISA for A*β* measurement was developed from day 14.5 to day 21 (12 h, 24 h, 48 h, 96 h, and 168 h after A*β* injection). Rat brains were removed for western blotting on day 21.

Two independent experiments were carried out in the present study. In the first experiment (Figure 1(a)), 30 rats were randomly divided into 3 groups for MWM test and HE as follows: sham group (10 rats, given orally 0.9% saline and injected 0.9% saline into brain), A*β* group (10 rats, given orally 0.9% saline and injected A*β*_42_ into brain), and A*β* + KXS group (10 rats, given orally KXS and injected A*β*_42_ into brain). On the 14^th^ day, A*β*_42_ or 0.9% saline were injected into rat brain; HE was developed at day 21 after MWM test from day 15 to day 20.

In the second experiment (Figure 1(b)), 120 rats were randomly divided into 3 groups for Enzyme-linked immunosorbent assay (ELISA) and western blot analysis. The groups, the treatment of drug-given, and A*β* injection were as same as that in the first experiment. However, A*β* measurement using ELISA was developed from day 14.5 to day 21 (12 h, 24 h, 48 h, 96 h, and 168 h after A*β* injection, 8 rats for each time point) to observe A*β* catabolic process in the brain of each rat, while western blotting was developed for protein expressions including NEP, ECE, and ACE at day 21. Moreover, from day 1 to day 21, the rats in the sham group and the A*β* group were orally given with 0.9% saline once a day, while the rats in the KXS group were orally given to KXS with a dosage of 1.08 g/kg.

### 2.3. KXS Extraction

KXS composition was the following: Ginseng, Poria, Polygalae, and Acorus. The four dried raw herbs were mixed together in a weight ratio of 3 : 3 : 2 : 2 (Ginseng = 60 g, Poria = 60 g, Polygalae = 40 g, and Acorus = 40 g), and decocted/extracted by refluxing for 1.5 h in 2000 ml boiling 60% ethanol (1 : 10, w/v). Then, the extracts were filtered, dried under vacuum, and stored at −80°C and the yield of KXS extracts was 20%.

### 2.4. A*β* Injection

A*β* injection was performed as follows: A*β*_42_ (Wako Pure Chemical Industries, Ltd., Japan) was dissolved in DMSO, diluted with 0.9% saline, and injected into rat hippocampus as previously described [8]. Briefly, rats were anesthetized and fixed on a stereotaxic instrument. A*β*_42_ (200 *μ*M, 1 *μ*L each side for a total of 2 *μ*L per rat) was accurately injected bilaterally into the skull above the dentate gyrus area of the hippocampus in 10 min. Five minutes after injection, the needle was slowly withdrawn from the brain, the surgical incision was sutured, and penicillin sodium was applied to prevent infection.

### 2.5. Morris Water Maze Test

The Morris water maze (MWM) test was used to confirm the effects of KXS on the cognitive function of the rats as previously described [9]. Briefly, MWM consisted of a black circular pool (diameter 120 cm, depth 40 cm) filled with water (25 ± 1°C). The pool was divided into four quadrants and a platform (12 cm in diameter) was placed in a quadrant of the pool, submerged 1 cm below the water surface. The rats were trained to find the platform in the pool, 5 trials per day (1 block, 90 s/trial) for 5 consecutive days. The time needed by the rats to search and reach the escape platform was counted as escape latency. On the 6^th^ day, the platform was removed and the rats have to search for the platform in the pool within 60 s; the swimming times across the platform area were recorded. The escape latency and the swimming times across the platform area were used to evaluate the memory function of each rat.

### 2.6. Histological Staining

After the behavioral experiment, the brain tissues of the rats in each group were removed and fixed in 4 % formalin for histological analysis. Rat brain was dehydrated and embedded in paraffin as previously described [10]. Histopathological changes were evaluated in 4 *μ*m thick deparaffinized brain tissue sections stained with Hematoxylin-Eosin (HE). Furthermore, the pathological neuronal injury was evaluated by neurons irregular shape, neurons arrangement, and neuronal death in different fields.

### 2.7. ELISA Study

At each time point (Figure 1(b)), the brain of each rat was removed and quickly dissected on ice and (left hemisphere of the brain was used for ELISA assay while the right hemisphere was used for western blot analysis) and left hemisphere of the brain was homogenized for 1 h in 70% formic acid buffer containing protease inhibitor cocktail (Roche, Switzerland). After the lysates were centrifuged at 100,000 ×*g* for 1 h, the supernatants were collected and neutralized with 1 M Tris-based solution. A*β*_42_ concentration was measured using a specific and sensitive Sandwich ELISA kit purchased from Wako Company (Japan) [8]. The bicinchoninic acid assay (BAC) method was used to measure total protein concentration. A*β*_42_ “Quantity-Time” curve was made on the basis of A*β*_42_ measurement results.

### 2.8. A*β* Degradation in PC12 Cells

PC12 cells were plated on 96-well dishes at a density of 1 × 10^4^ cells/well. PC12 cells were treated with 10% rat serum containing KXS (rat serum was collected after daily oral administration of KXS (1.08 g/kg) for three days) in the culture medium, thiorphan (10 *μ*M), or KXS plus thiorphan for 24 h, respectively. The control group was treated with 10% rat serum without KXS (rat serum was collected after daily oral administration of normal saline for three days). The culture medium was removed on the day of the experiment, and the monolayers were preincubated with A*β* (1 *μ*M) in the culture medium for 3 hours. The culture medium (100 *μ*L) contained in each well was then transferred to a plastic vial for quantitation by ELISA (BlueGene Biotech).

### 2.9. Western Blot Analysis

To detect NEP, ACE, and ECE, the hippocampi were dissociated and lysed in radioimmunoprecipitation assay (RIPA) buffer containing protease inhibitor for 20 minutes. Next, 10 *μ*L protein samples (2 *μ*g/*μ*L) were resolved on 10% sodium dodecyl sulfate polyacrylamide gel electrophoresis (SDS-PAGE) and transferred to polyvinylidene difluoride (PVDF) membranes. The membranes were blocked by 5% skim milk powder for 2 hours and subsequently incubated with primary antibodies against NEP (1 : 100), ACE (1 : 100), and ECE (1 : 400) overnight at 4°C. Immunoreactive bands were incubated with the HRP-linked secondary antibody (1 : 800) for 2 hours. After washing with tris-buffered saline (TBS) and Tween 20 (TBST), enhanced chemiluminescence (ECL) detection system (Beyotime) was used to quantify the optical densities of immunoreactive bands using Gel Imaging System (BIO-RAD, USA). All the protein bands were analyzed by comparison with the internal reference protein (*β*-actin).

### 2.10. Statistical Analysis

All data were expressed as mean ± SD. ANOVA was used for comparisons of multiple groups and *LSD-t* was used between two groups. *Ap* value less than 0.05 was considered statistically significant.

## 3. Results

### 3.1. KXS Improved Rat Cognitive Deficit Induced by A*β*_42_

**Figure 2:**
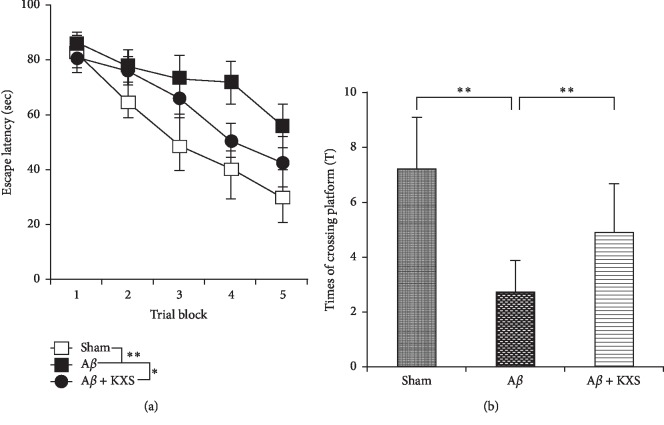
KXS prevented the cognitive deficit induced by A*β*42 injection. (a) In the navigation test, the average escape latency of each group changed gradually and became shorter following training during the 5 days. From the 3rd to 5th day, the average escape latency of the A*β* group was significantly longer than that in the sham group and A*β* + KXS group (^*∗∗*^*P* < 0.01, ^*∗*^*P* < 0.05; *n* = 10). (b) In the spatial probe test, the average times of the animals swimming across the platform area of the A*β* group were reduced significantly compared to that of the sham group. After the oral administration of KXS, the average times were significantly increased (^*∗∗*^*P* < 0.01, ^*∗*^*P* < 0.05; *n* = 10).

MWM test was used to confirm the effects of KXS on cognitive function. Significant differences were found in escape latency and the time of crossing platform area between the A*β* group and the sham group (*P* < 0.01), indicating that A*β* injection into the hippocampus caused cognitive deficits in the rats. In the animals that underwent KXS administration, however, memory dysfunction induced by A*β* was dramatically reduced. Indeed, in the place navigation test (Figure 2(a)), the escape latency of rats was in the A*β* + KXS group significantly shorter than that in the A*β* group (*P* < 0.01). In the spatial probe test (Figure 2(b)), the times of crossing the platform area increased significantly in the A*β* + KXS group compared to that in the A*β* group (*P* < 0.05). These results indicated that KXS improved rats' cognitive deficit induced by A*β*.

### 3.2. KXS Attenuated Hippocampal Neurons Injury

**Figure 3:**
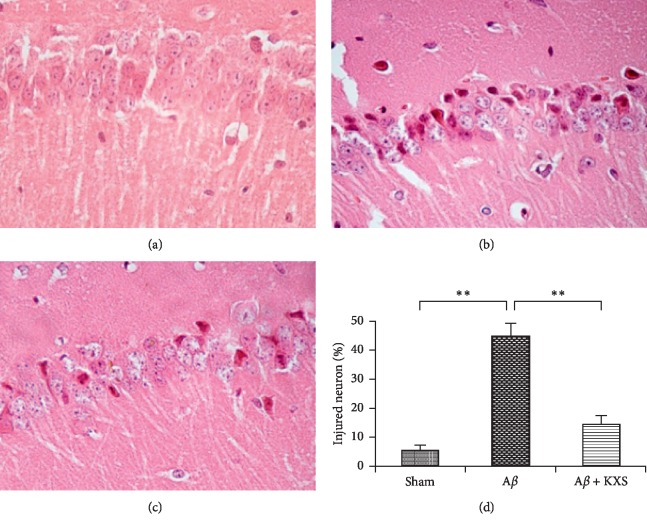
KXS prevented the neuronal pathological injury by A*β*42 injection. (a) Few damaged neurons in the sham group. (b) A neuronal pathological injury was induced by injected A*β*_42_. (c) The A*β* induced neuronal injury was ameliorated after treatment with KXS. (d) Statistic analysis of injured neurons. Light microscope slices in each of the 5 randomly selected fields, the number of injured neurons (irregular neuronal shape and arrangement) and dead neurons (disappearance of nucleolus) were recorded. The average proportion of injured neurons in the A*β* + KXS group was significantly reduced compared with that in the A*β* group (^*∗∗*^*P* < 0.01, *n* = 10).

HE stain was performed to analyze histological evidence explaining the above memory function changes (Figure 3). The results showed pathological neuronal injury such as irregular neuronal shape and arrangement, as well as neuronal death in the hippocampus after A*β* injection (Figure 3(b)). However, the damage was remarkably reduced in the A*β* + KXS group (Figures 3(c) and 3(d)), and the number of surviving neurons increased significantly compared to the A*β* group (*P* < 0.01, Figure 3(b)), suggesting that KXS could prevent neuronal injury induced by A*β*_42_.

### 3.3. KXS Decreased A*β*_42_ Concentration

**Figure 4:**
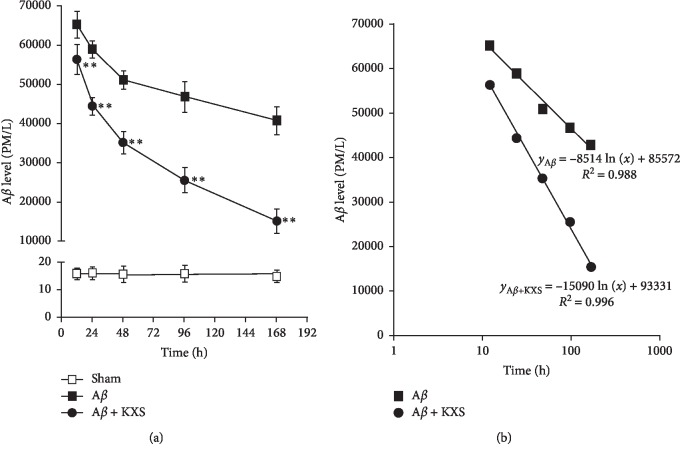
KXS decreased A*β*42 concentration after injection in the brain (a) A*β* levels were assessed by specific ELISA with the same time interval. At *t* = 0, rats were injected with A*β*_42_ directly into the hippocampus and the brains were sampled for A*β* measurement at *t* = 12, 24, 48, 96, 168 hours. As time went on, the injected A*β*_42_ gradually declined in each group (^*∗∗*^*P* < 0.01 vs. A*β* on identical time points; *n* = 8). (b) The curves of the logarithm model were used to fit the trend of A*β*_42_ degradation. By analysis of the parameters of the two regression equations (i.e., A*β* + KXS of 15090 > A*β* of 8514), which represent the ratio of A*β*_42_ degradation, we demonstrated that the A*β* concentration was accelerated after treatment of KXS.

The next step was to explore the mechanism conferring to the ability of KXS to reduce A*β* induced injury. A*β* levels in the hippocampus were gradually reduced after A*β* injection. A*β* levels were significantly lower in the A*β* + KXS group compared to that in the A*β* group at each point (*P* < 0.01, Figure 4(a)). Moreover, the experimental data were fitted well with the logarithm model to analyze the degradation ratio of A*β* (Figure 4(b)). By comparing the regression coefficients of two regression equations (15090 > 8514), we suggested that KXS decreased A*β*_42_ concentration.

### 3.4. KXS Upregulated the Expression of NEP but Not ACE and ECE

**Figure 5:**
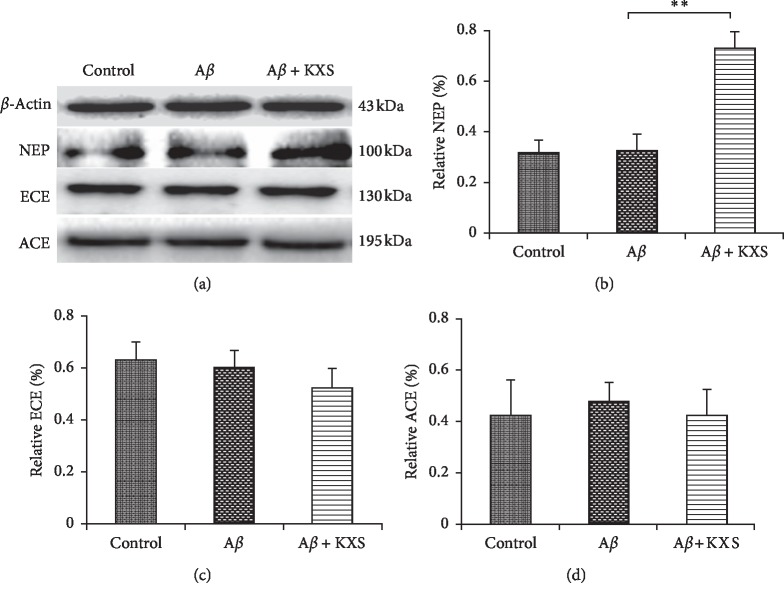
KXS upregulated the expression of NEP, but not ACE and ECE. (a) Protein expression of NEP, ACE, and ECE measured from the samples of the hippocampus of rats. (b) Compared to the A*β* group, the relative expression of NEP increased significantly in the A*β* + KXS group. No significant changes were found in the relative expression of ECE (c) and ACE (d), respectively (^*∗∗*^*P* < 0.01, *n* = 10).

To further explore the A*β* reduction mechanism due to KXS, three main ADEs protein expressions such as NEP, ACE, and ECE were investigated in hippocampal tissue by western blotting. NEP expression was significantly higher in the A*β* + KXS groups than that in the A*β* group (*P* < 0.01, Figures 5(a) and 5(b)). However, ACE and ECE protein levels were not changed in the KXS group and A*β* group (Figures 5(a), 5(c), and 5(d)). These results indicated that KXS could upregulate NEP expression in the brain.

### 3.5. KXS Decreased A*β* Concentration in PC12 Cells

**Figure 6:**
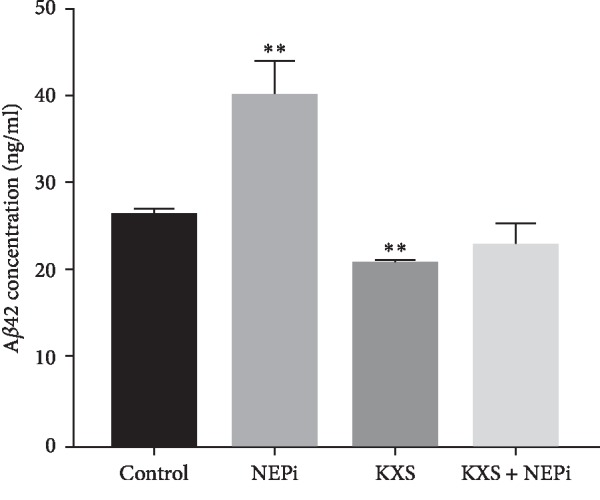
Effect of KXS on NEP activity in PC12 cells. PC12 cells were treated with 10% rat serum containing KXS in the culture medium, thiorphan (NEP inhibitor, 10 *μ*M), or KXS plus thiorphan for 24 h, respectively. Then PC12 cells were incubated with A*β* (1 *μ*M) for 3 h in the culture medium and extracellular A*β* concentration was investigated using the ELISA kit. Data were expressed as mean ± S.D (^*∗∗*^*P* < 0.01 vs. control; *n* = 3).

PC12 cells were employed to evaluate the effect of KXS on NEP activity *in vitro*. We examined the accumulation of A*β*. When pretreated with KXS in PC12 cells, extracellular concentration of A*β* was decreased by KXS in PC12 cell medium, while it was elevated by thiorphan (known NEP inhibitor, NEPi) (Figure 6). The *in vitro* results confirmed that KXS could elevate NEP activity, accelerating A*β* degradation.

## 4. Discussion

Lines of evidence supported that A*β*, especially its soluble oligomeric forms, causes a series of pathological changes including the dysfunction and loss of synapses [7], loss and impairment of neurons, cholinergic dysfunction, and aberrant neural network activity [1, 11]. Besides, the inflammatory and oxidative injury usually takes place after the formation of senile plaque. The copathogenic interactions among diverse factors were usually responsible for the cognitive disorder in AD development [1]. Thus, the excessive A*β* accumulation has been considered as the upstream factor in the cascade of events that characterized AD development [12]. Correlated with the toxicity of A*β*, many studies have shown that multiple forms of A*β* cause experimental dementia in animals and induce cell injury *in vitro* [13]. In the present study, behavioral tests by MWZ showed cognitive disorders induced by A*β* injection, while cognitive disorders were improved after KXS treatment. Furthermore, HE staining results also revealed the protective effect of KXS on hippocampal neurons. As a consequence of that, hippocampal neurons whose dysfunction and death due to A*β* was responsible for learning and memory impairment were protected by KXS, preventing the loss of memory. These results indicated that KXS prevented cognitive disorder and inhibited the injury of hippocampal neurons. Moreover, A*β* consecutive measurement showed that the injected A*β*_42_ was gradually reduced in the brain of both the A*β* group and A*β* + KXS group (Figure 4). “Quantity-Time” of A*β*_42_ showed that A*β* was reduced faster after KXS treatment, which can be analyzed by the parameters of two regression equations. The results also indicated that decreased A*β* concentration is not due to diffusion but due to A*β* degradation. However, many pathways participated in A*β* clearance through a combination of transport across vessel walls into the blood stream, diffusion along perivascular extracellular matrix and ADEs [14]. In our studies, we focused on the effect of KXS on ADEs mediated A*β* degradation, and western blotting was performed to detect ADEs expression. Whether KXS could affect other pathways of A*β* clearance, we will further explore in our further studies.

Studies have demonstrated that the normal content of A*β* in the brain mainly depends on the metabolic balance between its anabolism and catabolism [2]. The anabolic A*β* increase is rarely observed in the sporadic AD [4]. Little evidence is available to support that the increase of A*β* upon aging is preceding the A*β* deposition [2]. The reduction in the catabolic activity of ADEs might be a possible reason for the increasing concentration of A*β* in the brain. Therefore, it might be reasonable to hypothesize the upregulation of ADEs to explain A*β* reduction. Our previous study showed that IDE protein expression was upregulated in rats treated with KXS without affecting the IDE mRNA level. However, other ADEs expressions were not further investigated [8]. In this study, KXS increased NEP expression in the hippocampus but had no effect on ACE and ECE expressions. NEP, a type II membrane-associated peptidase, is exclusively expressed in neurons and more evidence proved that NEP is the predominant ADE for degrading both monomeric and oligomeric forms of A*β* [15]. It has been reported that NEP plays a critical role in inhibiting A*β* accumulation in animals [16]. Furthermore, NEP activity was activated by KXS in PC12 cells, leading to accelerating A*β* degradation. Due to the effect of NEP on degrading A*β*, we concluded that the upregulation of NEP expression after treatment of KXS in our study was one important factor for A*β*_42_ reduction.

## 5. Conclusion

In conclusion, KXS facilitated A*β*_42_ degradation through increasing NEP expression in the hippocampus. By reducing the A*β* burden, KXS protected hippocampal neurons and prevented the loss of cognitive function in the rats. Our findings not only clarified KXS molecular mechanism on protecting brain neurons from A*β* toxicity but also provided a new strategy for AD treatment.

## Data Availability

The data used to support the findings of this study are included within the article.
